# Characteristics and availability of medicine early access programs and donations in Slovenia

**DOI:** 10.1093/oncolo/oyaf092

**Published:** 2025-07-22

**Authors:** Lea Knez, Janja Jazbar, Mitja Kos

**Affiliations:** University of Ljubljana, Faculty of Pharmacy, 1000 Ljubljana, Slovenia; University Clinic Golnik, 4204 Golnik, Slovenia; University of Ljubljana, Faculty of Pharmacy, 1000 Ljubljana, Slovenia; University of Ljubljana, Faculty of Pharmacy, 1000 Ljubljana, Slovenia

**Keywords:** early access programs, compassionate use programs, antineoplastic and immunomodulating agents, oncology, access time

## Abstract

**Background:**

Early access programs (EAPs) and medicine donation programs (MDPs) enable patients to have access to new medicines prior to regulatory approval or national reimbursement, respectively. The objective of this study was to evaluate the characteristics and timeliness of EAPs and MDPs in Slovenia.

**Methods:**

Originator medicines approved by the EMA through a centralized procedure from 2010 to 2019 were included in the study. Data on the EAPs and MDPs were obtained from the Agency for Medicinal Products and Medical Devices of Slovenia.

**Results:**

The EMA approved 458 new indications for 324 medicines during the study period. In Slovenia, a total of 58 medicine indications (12.7%) became available before national reimbursement, including 35 (7.6%) by EAPs and 26 (5.7%) by MDPs (3 through both programs). Among these, 35 (60.3%) medicine indications were associated with oncology. EAPs facilitated access to medicines a median of 21.0 months before reimbursement (24.7 months for oncology medicines). Initiating EAPs at the time of the marketing authorization application could potentially improve the time of early access by an additional 5.5 months (5.2 months for oncology). MDPs enabled access to medicines 9.5 months prior to reimbursement (9.4 months for oncology), with potential further improvement by 7.9 months with the initiation of access at the time of marketing authorization (5.7 months for oncology).

**Conclusion:**

Our findings highlight Slovenia’s successful implementation of EAPs and MDPs, with oncology emerging as the predominant focal area. The potential exists for improvements in the scope, timeliness, and transparency of information on EAPs and MDPs accessible to patients in Slovenia.

**Implication for Practice**

Early access programs (EAPs) and medicine donation programs (MDPs) are especially important for patients with life-threatening conditions and exhausted treatment options, as many cancer patients. In Slovenia, a total of 58 of 458 evaluated medicines became available early, facilitating access to a medicine 21.0 (EAPs) and 9.5 months (MDPs) prior to national reimbursement. Nevertheless, the extent and time to access EAPs and MDPs may be further improved to benefit patients, healthcare systems, and society at large. This is most valuable in countries with limited access to clinical trials and long reimbursement procedures.

## Introduction

Patients can access a medicine once its benefits are proven to outweigh its potential risks and marketing authorization and negotiations for national reimbursement have been acquired.^1^ The traditional drug development pathway is often lengthy, necessitating years of clinical trials and regulatory approvals before innovative treatments are available to patients.^2^ The centralized marketing authorization procedure ensures EU-wide access to medicines through a unified process. However, access to medicines among member states can vary due to differences in the time required for reimbursement.^3^ Stringent evaluations are intended to provide effective and safe therapies for every patient, yet there is an unmet need for patients with serious conditions that have exhausted all appropriate authorized therapy.^3,4^ This concern is particularly evident for individuals with advanced-stage cancer who do not have the luxury of waiting for the approval of a new treatment.^5^

One option for these patients to gain early access to promising therapies is through participation in clinical trials.^5^ However, this avenue is restricted to patients meeting specific inclusion criteria, and it may be further limited in countries where clinical trials are rare, as in the case of Slovenia.^6^ An alternative option is the use of early access programs (EAPs) that provide unregistered medicines to patients when no other treatment options are available.^4^ These programs serve patients facing life-threatening or seriously debilitating conditions who have no options for comparable or satisfactory alternative authorized therapy.^1^ The medicine must be undergoing clinical trials or have marketing authorization pending.^7^

Early access programs operate under national regulations in each member state, only guided by general European Medicines Agency (EMA) recommendations.^1,7^ In Slovenia, EAPs have been established to facilitate access to medicines before marketing authorization.^8^ Additionally, another viable option available in Slovenia is through medicine donation programs (MDPs), which enable access to medicines after marketing authorization but prior to reimbursement.^8^

Despite the presence of EAPs in most EU member states,^4^ understanding of the extent and characteristics of implemented EAPs remains limited. Moreover, individual countries may demonstrate some variability in EAPs due to their specific national legislation. Previous research has described EAPs in specific pharmacotherapeutic areas^9,10^ and explored their structure and processes across different countries.^3,4,11,12^ However, extensive studies evaluating the time needed to access medicines through EAPs at the national level are scarce.^3^ Thus, the primary objective of this study was to evaluate the main characteristics and availability of EAPs and MDPs in Slovenia, focusing on the number of available programs, the time of early access, and the time of potential additional early access.

## Methods

### Medicine selection

All originator medicines (reference medicinal products) that were approved for either a first or subsequent indication by the EMA through a centralized procedure during the period from 2010 to 2019 were selected for the study. Each indication of a given medicine was analyzed independently as a distinct entity termed a “medicine-indication.”

### Definition of EAP and MDP

**Figure 1.**
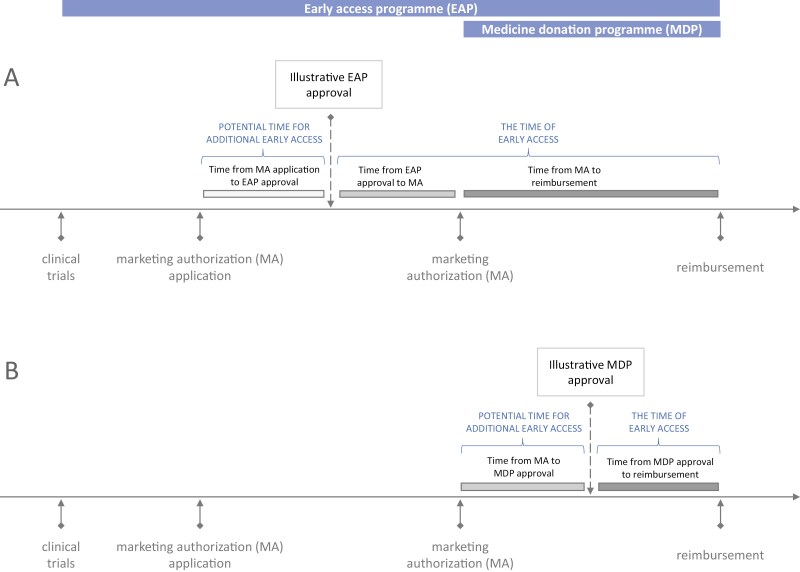
The timeline and the definition of time variables for EAPs (**A**) and MDPs (**B**).

Herein, early access to medicines refers to the patient’s access to a medicine before the date of reimbursement for a specific medicine indication. In Slovenia, two distinct procedures facilitate such access (Figure 1).^8^ The first procedure encompasses EAPs, which enable patients to gain access to medicines before their marketing authorization. Slovenian legislation does not distinguish between compassionate use programs and named patient programs. The second program facilitating early access is the MDP, which allows patients access to medicines after marketing authorization, but typically before reimbursement is granted. These programs differ in the eligibility criteria for medicines. EAPs are restricted to treatments for life-threatening or seriously debilitating conditions, whereas MDPs have no such limitations. Both programs are initiated and fully funded by the holder of the marketing authorization of a medicine, although the need for a medicine is usually prompted by healthcare professionals. The processing of applications for both programs is overseen by the Agency for Medicinal Products and Medical Devices of the Republic of Slovenia (JAZMP). All EAPs have to be approved by JAZMP, and since 2014 all MDPs have to be reported to the agency. The collection of real-world data from patients enrolled in these programs is not mandated by JAZMP but may be conducted separately as an independent activity. The present study evaluated both the EAPs and the MDPs.

### Data sources

Comprehensive information on marketing authorization procedures and their respective dates (application and approval dates) were obtained from the official EMA website.^13^ Data on the availability and approval dates of EAPs and MDPs in Slovenia are not publicly available and were obtained by JAZMP upon request. For each EAP or MDP, JAZMP maintains records on the medicine, its indication, end-users, and approval date. However, patient-level data are not available. For the purpose of the analysis, all EAPs and MDPs approved for medicine-indications under study were selected. Information on medicine reimbursement dates was obtained until the end of the year 2020 from the Health Insurance Institute of Slovenia, the nationwide Bismarck-type obligatory health insurance institute.^14^

### Outcomes

Three key aspects were evaluated. First, the number and characteristics of available programs were considered, including the EAPs and MDPs implemented in Slovenia during the study period, in total, and by anatomical therapeutic chemical (ATC) codes. Second, the time of early access in each program was defined as the duration between the approval of the respective program and the actual medicine reimbursement (Figure 1). For EAPs available before the marketing authorization application, the time between the marketing authorization application and EAP approval was set to zero. Third, the potential time for additional early access in EAPs was identified as the interval between marketing authorization application and EAP approval, while for MDPs, it was determined as the period between marketing authorization and MDP approval. The EAP and MDP end date was defined as the date of reimbursement or the end of 2020 if reimbursement did not occur before then (10 medicine-indications). The time zero was defined as the date of the EAP or MDP approval.

The characteristics of EAPs and MDPs were summarized using descriptive statistics, presenting information on overall sample size, year of access, ATC codes, and end-users of the programs. To illustrate the time of access, graphical representations were employed, complemented by descriptive statistical analyses.

## Results

### Number of EAPs and MDPs

During the period spanning 2010 to 2019, the EMA approved a total of 458 new medicine indications for 324 distinct medicines (Table 1). In Slovenia, a total of 58 medicine-indications (12.7%) were available prior to their reimbursement. Among these, 35 (7.6%) were accessible through EAPs, while 26 (5.7%) were made available via MDPs; notably, 3 medicine indications were accessible through both EAPs and MDPs. Antineoplastic and immunomodulating agents were primarily available through EAPs, while other medicines were mostly available through MDPs. Among the total of 58 medicine indications included in EAPs or MDPs, the majority, 37 (63.8%), belonged to the class of antineoplastic and immunomodulating agents (ATC L), while 10 (17.2%) were categorized as anti-infectives for systemic use (ATC J), and four (6.9%) belonged to cardiovascular medicines (ATC C).

**Table 1.** Characteristics of medicine-indications within EAPs and MDPs relative to all medicine-indications approved by the EMA in a centralized procedure between 2010 and 2019.

	Medicine indications between 2010 and 2019			
Characteristic	Available through EAPs	Available through MDPs	Available through EAPs or MDPs	Total: all approved by EMA
Number (% of total)	35 (7.6)	26 (5.7)	58 (12.7)	458 (100)
ATC level 1; *N* (% of total)				
A, alimentary tract and metabolism	0	2 (4.9)	2 (4.9)	41 (100)
C, cardiovascular system	1 (5.3)	4 (21.1)	4 (21.1)	19 (100)
H, systemic hormonal preparations	1 (20.0)	0	1 (20.0)	5 (100)
J, anti-infectives for systemic use	3 (5.7)	7 (13.2)	10 (18.9)	53 (100)
L, antineoplastic and immunomodulating agents	29 (15.1)	9 (4.7)	37 (19.3)	192 (100)
M, musculo-skeletal system	1 (11.1)	1 (11.1)	1 (11.1)	9 (100)
N, nervous system	0	1 (3.7)	1 (3.7)	27 (100)
R, respiratory system	0	1 (4.0)	1 (4.0)	25 (100)
V, various	0	1 (5.9)	1 (5.9)	17 (100)
Other (ATC B, D, G, P, S)	0	0	0	70 (100)
Oncology field; *N* (% of total)				
Total	27 (17.6)	9 (5.9)	35 (22.9)	153 (100)
Non-solid tumours	9 (17.3)	4 (7.7)	13 (25.0)	52 (100)
Solid tumours	18 (17.8)	5 (5.0)	22 (21.8)	101 (100)
Lung cancer	8 (25.0)	3 (10.7)	9 (32.1)	28 (100)
Prostate cancer	4 (50.0)	0	4 (50.0)	8 (100)
Skin cancer	3 (15.0)	1 (5.0)	4 (20.0)	20 (100)
Breast cancer	3 (27.3)	0	3 (27.3)	11 (100)
Reproductive system cancer	0	1 (25.0)	1 (25.0)	4 (100)
Other	0	0	0	40 (100)

Patients in Slovenia received medicines through EAPs and MDPs via secondary health centers (hospitals) or tertiary health centers (university medical centers and institutes). Since novel medicines, especially for oncology and other life-threatening or seriously debilitating conditions, are primarily introduced in secondary or tertiary healthcare settings, it was expected that no programs would be available in primary care centers. A substantial proportion of both secondary and tertiary health centers in Slovenia have integrated EAPs or MDPs, with 57.1% (12 out of 21) of hospitals and 66.6% (4 out of 6) of tertiary health centers having at least one program available.

### Characteristics of EAPs and MDPs

Within 58 medicine indications, 35 medicine indications (60.3%) were associated with oncology (Table 2). Among these 35 medicine indications, 22 (62.9%) were indicated for solid tumors, while the remaining 13 (37.1%) were indicated for non-solid tumors. The specific types of solid tumors covered by the EAPs and MDPs included lung cancer (9 indications), prostate cancer (4 indications), skin cancer (4 indications), breast cancer (3 indications), and reproductive system cancer (1 indication). The majority of EAPs (77.1%) belonged to oncology, whereas among MDPs, only one-third (34.6%) were oncology medicine-indications.

**Table 2.** Characteristics of medicine-indications from oncology relative to all medicine-indications within EAPs or MDPs.

	Medicine indications between 2010 and 2019			
Characteristic	Available through EAPs	Available through MDPs	Available through EAPs or MDPs	Total: all approved by EMA
All medicine-indications				
Total number	35	26	58	458
Oncology medicine-indications				
Total oncology; *N (% relative to total number)*	27 (77.1)	9 (34.6)	35 (60.3)	153 (33.4)
Non-solid tumours; *N (% relative to total oncology)*	9 (33.3)	4 (44.4)	13 (37.1)	52 (34.0)
Solid tumours; *N (% relative to total oncology)*	18 (66.7)	5 (55.6)	22 (62.9)	101 (66.0)
Lung cancer	8 (29.6)	3 (33.3)	9 (25.7)	28 (18.3)
Prostate cancer	4 (14.8)	0	4 (11.4)	8 (5.2)
Skin cancer	3 (11.1)	1 (11.1)	4 (11.4)	20 (13.1)
Breast cancer	3 (11.1)	0	3 (8.6)	11 (7.2)
Reproductive system cancer	0	1 (11.1)	1 (2.9)	4 (2.6)

### The time of early access for medicines within EAPs and MDPs

**Figure 2.**
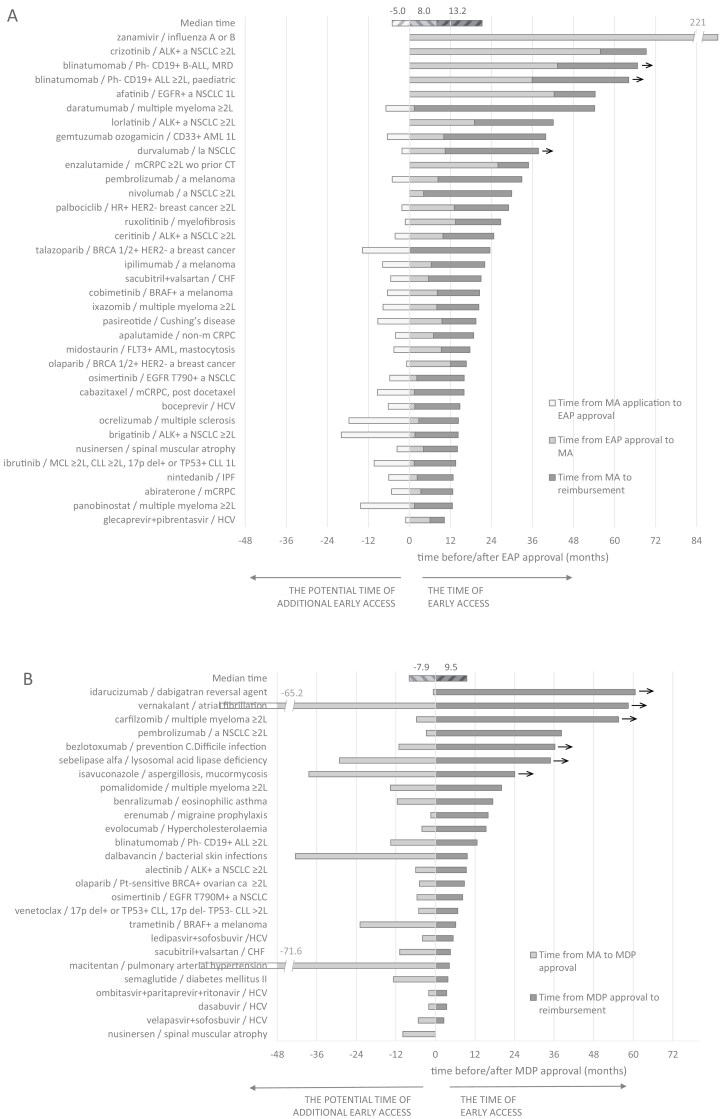
The time of early access (in months) and the potential time of additional early access for medicine indications available through EAPs (A) and MDPs (B) in Slovenia between 2010 and 2019 and median time of access. The zero presents the time of the EAP (A) or MDP (B) approval. The arrows indicate medicines that were still waiting for reimbursement at the end of year 2020. Abbreviations: ≥2L, relapsed or refractory to first-line treatment; 17p del, 17p deletion; 1L, treatment naive; a, advanced; ALK, anaplastic lymphoma kinase; ALL, acute lymphoblastic leukaemia; AML, acute myeloid leukaemia; B-ALL, B-precursor acute lymphoblastic leukaemia; BRCA, breast cancer gene; C.Difficile, Clostridium difficile; ca, cancer; CHF, chronic heart failure; CLL, chronic lymphocytic leukaemia; EAP, early access programme; EGFR, epidermal growth factor receptor; FLT3, fms-related receptor tyrosine kinase 3; HCV, hepatitis C virus; HER2, human epidermal growth factor receptor 2; HR, hormone receptor; IPF, idiopathic pulmonary fibrosis; la, locally advanced; MA, marketing authorization; MCL, mantle cell lymphoma; mCRPC, metastatic castration-resistant prostate cancer; MDP, medicine donation programme; MRD, minimal residual disease; non-m CRPC, non-metastatic castration-resistant prostate cancer; NSCLC, non-small-cell lung cancer; Ph, Philadelphia chromosome; Pt-sensitive, platinum sensitive; wo prior CT, without prior chemotherapy.

Among the 35 EAPs, medicine indications were available 21.0 (15.4-35.3) months (median time, Q1-Q3) before medicine reimbursement (Table 3, Figure 2). Within this timeframe, medicine indications were available 6.4 (1.9-10.3) months prior to marketing authorization, with an additional 13.9 (11.6-23.5) months before reimbursement. A majority of EAPs, 29 (82.9%) became accessible after the marketing authorization application, while the remaining six (17.1%) were made available before the marketing authorization application. The time of early access was longer than 2 years for 15 (42.9%) medicine indications and longer than 1 year for 34 (97.1%) medicine indications (Figure 2A). Notably, if all 29 EAPs initiated after the marketing authorization application had been instead initiated at the time of marketing authorization submission, the time of early access could potentially have been improved by an additional 5.5 (1.7-8.5) months (the potential time of additional early access). Within oncology medicine indications, the time of early access was 24.7 (17.2-38.7) months, and the potential time of additional early access was 5.2 (1.7-8.6) months.

**Table 3.** Timelines (Median; Q1-Q3) of EAPs or MDPs from the oncology field relative to all medicine indications.

	Medicine indications between 2010 and 2019	
Characteristic	Available through EAPs	Available through MDPs
All medicines		
Potential time of additional early access; *median (Q1-Q3)*	5.5 (1.7 to 8.5)	7.9 (4.3 to 13.6)
The time of early access altogether; *median (Q1-Q3)*	21.0 (15.4 to 35.3)	9.5 (4.7 to 23.1)
* Time to marketing authorization*	*6.4 (1.9 to 10.3)*	
* Time to reimbursement*	*13.9 (11.6 to 23.5)*	*9.5 (4.7 to 23.1)*
Oncology medicines		
Total oncology		
Potential time of additional early access; *median (Q1-Q3)*	5.2 (1.7 to 8.6)	5.7 (5.1 to 13.6)
The time of early access altogether; *median (Q1-Q3)*	24.7 (17.2 to 38.7)	9.4 (8.3 to 20.1)
* Time to marketing authorization*	*8.0 (1.6 to 11.3)*	*/*
* Time to reimbursement*	*14.9 (12.2 to 25.1)*	*9.4 (8.3 to 20.1)*
Non-solid tumours		
Potential time of additional early access; *median (Q1-Q3)*	6.5 (1.2 to 7.7)	9.7 (5.6 to 13.6)
The time of early access; *median (Q1-Q3)*	26.7 (17.7 to 53.1)	16.4 (11.2 to 28.9)
* Time to marketing authorization*	*9.3 (1.5 to 13.5)*	*/*
* Time to reimbursement*	*13.2 (12.1 to 28.2)*	*16.4 (11.2 to 28.9)*
Solid tumours		
Potential time of additional early access; *median (Q1-Q3)*	5.1 (2.2 to 9.0)	5.6 (4.9 to 6.0)
The time of early access; *median (Q1-Q3)*	24.1 (17.2 to 32.1)	8.8 (8.3 to 9.4)
* Time to marketing authorization*	*6.7 (1.8 to 10.3)*	*/*
* Time to reimbursement*	*15.3 (12.4 to 24.3)*	*8.8 (8.3 to 9.4)*

Regarding MDPs, these programs enabled access to medicines a median of 9.5 (4.7-23.1) months before reimbursement. The time of early access was longer than 2 years for seven (26.9%) medicine indications and longer than 1 year for 12 (46.2%) medicine indications (Figure 2B). However, if MDPs had been initiated at the time of marketing authorization, the time of early access could have potentially been improved by an additional 7.9 (4.3-13.6) months (the potential time of additional early access). Within oncology medicine indications, the time of early access was 9.4 (8.3-20.1) months, and the potential time of additional early access was 5.7 (5.1-13.6) months.

## Discussion

Our study describes the main characteristics and timeliness of programs facilitating patients’ access to medicines prior to regulatory approval and national reimbursement, on a country level and for all medicines approved by the EMA between 2010 to 2019. The findings highlight Slovenia’s successful implementation of such programmes in the form of EAPs and MDPs that provided early access to a total of 58 medicine indications, with oncology emerging as the predominant focal area. The EAPs and MDPs enabled patients’ access to medicines substantially earlier, 21.0 and 9.5 months, respectively, prior to reimbursement for the medicines. Streamlining the process of EAPs and MDPs could potentially increase early access by an additional 5.5 and 7.9 months, respectively.

The inclusion of 58 medicine indications within EAPs or MDPs represents 12.7% of all registered indications for originator medicines during the specified period. This finding indicates that, in Slovenia, the regulations and policies for EAPs and MDPs are established, fulfilling the first prerequisite to allow early access to medicines. The majority of the 58 medicines (60.3%) accessed early were for an oncology indication, which would be expected as cancer is often a life-threatening condition for which treatment options are, sooner or later, exhausted. Similar observations were made when the US Food and Drug Administration’s (FDA’s) expanded access and compassionate use programs were analyzed.^15^ Within the oncology cohort, a majority of indications targeted solid tumors (62.9%), including lung, prostate, skin, breast, and reproductive system cancers, mirroring the significant medicine development in recent years.^3,5,16^ The higher adoption of EAPs and MDPs in this specific area may be partly attributed to the increased awareness of these programs among oncologists, who subsequently prompt the marketing authorization holder to initiate them. Despite these promising findings, these early accessed medicines represent only a fifth (22.9%) of all oncology indications approved within the 10-year observational period. A study conducted in France by Jacquet et al.^3^ reported a significantly higher proportion, revealing that more than half (56.2%) of oncology medicines were accessible through the French program for temporary authorization for use before the formal inclusion of the medicines in the healthcare system. While direct comparisons between our study and the French study are not feasible, this finding suggests the potential for further improvement in Slovenia’s early access initiatives.

In Slovenia, medicine access is characterized by a fairly good rate of available medicines, as approximately half of all medicines and oncology medicines that gained EMA marketing approval between 2018 and 2021 were reimbursed by the beginning of 2023 (72/168 and 23/46, respectively).^17^ However, the median times from EMA approval to inclusion into the healthcare system exceed 400 days, with specific delays of 502 and 403 days for all medicines and oncology medicines, respectively. This lag time is indeed too long for patients with a life-threatening disease who have exhausted treatment options; thus, EAPs to medicines are important to fill this gap. This was also proven in our study, in which EAPs and MDPs enabled access to medicines 21 and 9.5 months prior to their reimbursement, respectively. These times were even longer for oncology medicines within EAPs (25 months). It is not surprising that oncology drugs were more often available through EAPs than MDPs, as the definitions of EAPs and MDPs imply the longer times observed with EAPs. In fact, EAPs but not MDPs may start prior to a medicine being granted a marketing authorization. In our study, they were started 6.4 months prior to EMA approval. This time is shorter than times reported for the US, with EAPs initiated 10.0 months prior to FDA approval, and in France, with temporary authorization for use starting 428 days (14 months) prior to EMA approval.^3,15^ Our finding is informative for all EU (and allied) countries, that share the same waiting time of over a year^2^ from a medicine application for marketing authorization to being granted the authorization and is possibly even more vital in countries such as Slovenia that have limited access to clinical trials.^6^ Moreover, both EAPs and MDPs bridge the gap from a medicine being approved to being reimbursed. In our study, patients were able to benefit from medicine at 13.9 and 9.5 months, respectively, prior to its inclusion in the healthcare system. This finding is indeed most interesting for countries with long reimbursement procedures, such as many Eastern and South-Eastern EU countries.^17-20^ The availability of programs facilitating access to medicines prior to their reimbursement is indeed important for all patients with a life-threatening disease and no viable treatment option, but it holds even more relevance in the case of essential medicines and pharmaceuticals that have entirely novel mechanisms of action (ie, “first-in-class”). Our data show that EAPs and MDPs allowed early access to some of the essential medicines that the World Health Organization identified as essential cancer medicines,^21^ including, eg, immune checkpoint inhibitors such as nivolumab and pembrolizumab, hormonal medicines such as abiraterone and enzalutamide, and targeted agents such as afatinib. In addition to these essential medicines, some first-in-class agents were also available, including palbociclib, olaparib, sacubutril, nusinersen, and boceprevir. As EAPs allow earlier access to medicines, these should be preferred over MDPs.

Our study highlights the potential for further optimization of the time of access through strategic program initiation. Initiating EAPs at the time of marketing authorization application could potentially improve the time of early access by an additional 5.5 months. In this regard, it is important to complement the start of many^8^ EAPs at the time or even preceding the application for marketing authorization, possibly in the form of named patient programs. Similarly, for MDPs, initiation at the time of marketing authorization could extend early access by an additional 7.9 months. These results offer a critical window of opportunity for improvements that should address all stakeholders. Patient and physician awareness should be raised, especially outside the oncology field.^5,16^ Regulatory bodies, manufacturers, and clinicians should identify ways to smooth early access procedures to diminish their administrative burden but still safeguard patient welfare. In fact, it is crucial to address the uncertainty on the risk-benefit ratio of medicines in EAP programs, for which assessment by regulatory bodies are still underway and even more when they are initiated prior to MA application, by implementing rigorous and transparent planning and data collection. Improving transparency on available programs, with details on inclusion and exclusion criteria, dates of initiation, and conclusion, possibly following the practice in the United States, should be mandatory, not only on a national but on a European level.^15^ In fact, although our study employed reliable data sources to gather comprehensive information on available EAPs and MDPs in Slovenia, these data were not publicly available and were limited in content. As our dataset lacked the date of program conclusion, it was assumed that EAPs and MDPs were available until a medicine gained reimbursement, possibly leading to an overestimation of the benefited time.

Overall, this study provides valuable insights into the landscape of EAPs and MDPs and their contribution to facilitating timely access to innovative medicines. By addressing the specific needs of patients, especially in oncology, and by optimizing the timing of program initiation, healthcare authorities, and stakeholders can further enhance the impact of these programs in supporting patient care and improving treatment outcomes. Future research should evaluate the contribution of programs for early access to medicines to patient outcomes and healthcare systems and society at large.^22,23^

## Data Availability

The data underlying this article will be shared on reasonable request to the corresponding author.
